# Routine data registries as a basis to analyse and improve the quality of antimicrobial prescription in primary care

**DOI:** 10.1186/s12875-025-03008-4

**Published:** 2025-10-17

**Authors:** Martijn Sijbom, Mirte Boelens, Mark G.J. de Boer, Mattijs E. Numans

**Affiliations:** 1 Department of Public Health and Primary Care / Health Campus The Hague, Leiden University Medical Centre, Turfmarkt 99, The Hague, Leiden, DP 2511 The Netherlands; 2 Department of Infectious Diseases, Leiden University Medical Centre, Leiden, The Netherlands; 3 Department of Clinical Epidemiology, Leiden University Medical Centre, Leiden, The Netherlands

**Keywords:** Primary care, Antimicrobial resistance, Antimicrobial stewardship, Determinants, Antimicrobial prescription, Healthcare registries, Data driven healthcare

## Abstract

**Background:**

The overuse of antimicrobials is the main driver of the increasing antimicrobial resistance (AMR). Between 80 and 90% of antimicrobial prescriptions originate from primary care. The goals were to establish the quality of prescriptions through combining data from a primary healthcare registry and a national socioeconomic database for novel determinants.

**Methods:**

Pseudonymized routine healthcare data from 269,547 patients (1,150,252 antimicrobial prescriptions) obtained between 2012 and 2020 from primary care practices (*n* = 101) in the region The Hague – Leiden were used. These data were linked with individual socioeconomic data from the Statistic Netherlands database to identify determinants of inappropriate antimicrobial prescribing. The quality of prescription was analysed using predefined criteria based on primary care guidelines. Multivariable logistic regression analyses were performed to identify associations with appropriateness.

**Results:**

Respiratory tract infections (RTI) were most commonly associated with inappropriate antibiotic prescribing, with 14.5% RTI prescriptions being inappropriate. For macrolide prescriptions, 77.1% did not correspond with 1st and 2nd guideline choices. Patient characteristics (migration backgrounds, female gender, comorbidities and age) and medium and large primary care practice size, a proxy for continuity of care and consultation time per patient, were associated with poorer guideline adherence.

**Conclusions:**

Combined analyses of socioeconomic and routinely collected healthcare data does reveal relevant additional information to answer medical questions in a broader context, such as AMR. Most room for improvement was found for RTIs and macrolides, especially in specific risk groups. Assuring continuity of care and/or providing extended consultation time per patient might be essential elements to establish, before disseminating improvement strategies.

**Supplementary Information:**

The online version contains supplementary material available at 10.1186/s12875-025-03008-4.

## Background

Antimicrobial resistance (AMR) is increasing worldwide and is a major threat to global health [1]. The leading driver of AMR is the use of antimicrobials [2]. Between 80 and 90% of antimicrobials for use in humans is prescribed in primary care [3]. Although development of multiresistant bacteria and other consequences of AMR occur mainly in hospitals, the role of primary care as the source of the increase in AMR is larger than previously assumed, presumably through antimicrobial selection pressure in the wider population [4]. Improving the quality of antimicrobial prescription in primary care may play an important part in avoiding further increase of AMR.

Although the number of antimicrobial prescriptions in the Netherlands is low compared to most other European countries [5], AMR has even increased in the Netherlands over the last 10 years [6]. To improve prudent antimicrobial prescribing, we need to identify determinants of (in)appropriate antimicrobial prescribing on patient and practice level. These determinants may then allow us to define specific risk groups and to identify specific elements in a primary care practice (PCP) that might be the target of antimicrobial stewardship interventions. However, information on socioeconomic context and PCP characteristics as potential determinants is lacking.

Healthcare registries harbouring routinely collected healthcare data, such as electronic medical records (EMR) from PCPs, are increasingly made available for research purposes. Combining those with large public dataset sources containing socioeconomic information, do arise to create new opportunities for AMR research. There is limited understanding of how large healthcare registries of routinely collected data can be combined and used in AMR research.

The aim of our current study was to identify patient - and practice associated determinants of antimicrobial prescribing in concordance with primary care guidelines and cues for further improvement.

## Methods

### Study design and setting

In this observational study, we analysed antimicrobial prescriptions for acute infections in primary care during weekdays between 8.00 and 17.00 for appropriateness, based on a large set of routine healthcare data combined with socioeconomic data from Statistics Netherlands (SN) over a period of ten years. The study was approved by the Medical Ethical Committee of Leiden University Medical Centre (LUMC) (file G20.020).

### Data collection through combining two large registries

This study used pseudonymized routine healthcare data derived from a data registry covering EMR data from approximately 450,000 patients. Patient EMR data registered from 2012 to 2020 were extracted from 115 PCPs affiliated with the Extramural LUMC Academic Network (ELAN), located in the Leiden-The Hague area of the Netherlands. ELAN covers 2.6% of the general Dutch population, and previous studies have established that data from ELAN are well generalizable to the average Dutch population [7, 8]. PCPs involved in ELAN provide continuous access to the pseudonymized EMR data of their practice population. An informed patient opt-out procedure concerning use of pseudonymized data for research is in place. The ELAN datawarehouse only contains data from patients consulting GP practices during office hours. Using data from the ELAN datawarehouse, the comorbidities associated with immunosuppression (Supplement 1) and antimicrobial allergies of each patient were linked to each antimicrobial prescription.

Oral antimicrobial prescriptions in the ELAN datawarehouse were identified through Anatomical Therapeutic Chemical (ATC) code J01. All prescriptions with ATC code J01 primarily prescribed by a PCP between 2012 and 2021 were included. International Classification of Primary Care (ICPC) codes (version 1) included with the prescription were used to define the reason for prescribing the antimicrobial. SN collects data on individual Dutch inhabitants (www.cbs.nl), they concern household income, migration background and number of parents in each household and were linked on a pseudonymized individual level. More details on the ELAN Datawarehouse and the linking process are described in a previous study [8].

### Data analysis

Antimicrobial prescriptions were examined for appropriateness, which was defined as a prescription in accordance with prevailing Dutch primary care guidelines at the time of prescription (Supplement 2) [9]. Prescribing an antimicrobial was considered inappropriate only if the recommendations advised against prescription. Antimicrobial prescriptions with an ICPC code corresponding with an infection were included in the analysis on appropriateness. An antimicrobial prescription was considered appropriate if the ICPC code accompanying the prescription matched an indication for an antimicrobial prescription in Dutch primary care guidelines. Prophylactic antimicrobial prescriptions with the intention to prevent infections were excluded, as the aim was to examine antimicrobial prescribing for acute infections. If the ICPC code was missing or obviously registered incorrectly, for example for hypertension, the antimicrobial prescription was excluded from the examination on appropriateness and further analysis. In a separate analysis, the choice of an antimicrobial corresponding to the 1st or 2nd choice antimicrobial in the prevailing guideline was viewed as corresponding to the guideline and therefore appropriate (Supplement 3). In case of a presumed antimicrobial allergy, Dutch primary care guidelines recommend a 3rd choice. If a patient had an antimicrobial allergy registration for the 1st and/or 2nd choice antimicrobial, the prescription of this 3rd choice was classified as corresponding to the guideline.

Primary outcomes were the number of appropriate and inappropriate antimicrobial prescriptions per year over the period 2012–2020. In the ELAN Datawarehouse, we identified 1,417,223 unique oral antimicrobial prescriptions by all PCPs (Supplement 4), of which 122,659 (8.2%) were identified as prophylaxis and subsequently excluded from further analysis. As SN had no data available for 35,321 patients (with 144,312 antimicrobial prescriptions), these prescriptions were also excluded. In total, 1,150,252 antimicrobial prescriptions for 269,574 unique patients were included in the analysis, (supplement 4).

### Determinants

A systematic literature review was conducted to identify determinants associated with appropriate antimicrobial prescribing [10]. Following that review, other potential determinants not yet investigated were defined, including migration background, household income, number of parents per household if there are children present and day of antimicrobial prescription.

#### Patient level

Included determinants on patient level were age, gender, comorbidity, migration background, household income and number of parents in household. Comorbidities that implied an immunosuppressed state, as listed in supplement 1, were merged into a composite comorbidity variable. Therefore, no analysis was done on comorbidities not associated with immunosuppression. For the calculation of this composite variable the presence of each comorbidity was counted as 1, added together as a count variable. We defined 4 comorbidity categories: 0, 1, 2 and 3 or more, and defined patients with 3 or more comorbidities as 1 group.

Household income was divided into 3 groups based on the definition of the Dutch Standardized income [11], 33,500 euro per year between 2012 and 2022. Our low income group had a household income of < 33,500 euro, middle income between 33,500 and 67,000 euros and high income group income of > 67,000 euro. Migration background was defined by SN as the country with which a person is connected based on the country of birth of one’s parents or oneself [12]. Migration background was categorized into seven groups according to SN definitions: Dutch, Dutch-Caribbean, Moroccan, Surinamese, Turkish and Global South and Global North. Number of parents in household was classified as a dichotomous variable of either one or two parents.

#### Practice and healthcare organisational level

Included determinants on primary care practice level were practice population size and day of prescription. During the study period, a PCP size of 2,168 patients was defined as the norm for a standard practice in the Netherlands by the Dutch Healthcare Authority [13]. As a general guideline, a standard practice can be managed by 1 full-time GP. For the analyses, PCPs were categorized into 3 groups according to the average size of their practice. A small practice was defined for our study as < 2,168 registered patients (i.e. smaller than the standard practice size), a medium size practice between 2,186 and 4,336 registered patients, and a large practice > 4,336 registered patients. PCPs were defined as outliers if the number of antimicrobial prescriptions was lower than 120 or higher than 750 antimicrobial prescriptions per 1000 patients per year. These outliers were attributed to incomplete EMRs. Data from these practices were not used in the final multivariable regression analyses. Day of prescription was divided into Monday-Thursday or Friday and was categorized as dichotomous.

### Statistical analyses

Paired sample t-tests were performed to test for statistically significant differences (*p* < 0,05) between number of antimicrobial prescriptions per year and the day of antimicrobial prescribing. Multivariable logistic regression analyses were performed to examine potential associations of the determinants with appropriate antimicrobial prescribing using four different models. Model 1 included gender (ref = female) and age (ref = 0–4 years). Model 2 additionally included migration background (ref = Dutch). Model 3 added number of parents in household (ref = 2 parents), household income (ref = low income) and number of comorbidities (ref = 0 comorbidities). Model 4 additionally included size of PCP (ref = small size) and day of prescription (ref = Friday). A multivariable logistic regression analysis using model four was conducted to examine possible determinants associated with appropriate antimicrobial prescribing for only respiratory tract infections (RTI). To check for possible bias due to missing data in SN database, a multivariable regression analysis was conducted that included patients with data from the ELAN datawarehouse but not registered in the SN database. No imputation of missing data was made, nor were additional categories employed.

## Results

### Trend of antimicrobial prescriptions

**Fig. 1:**
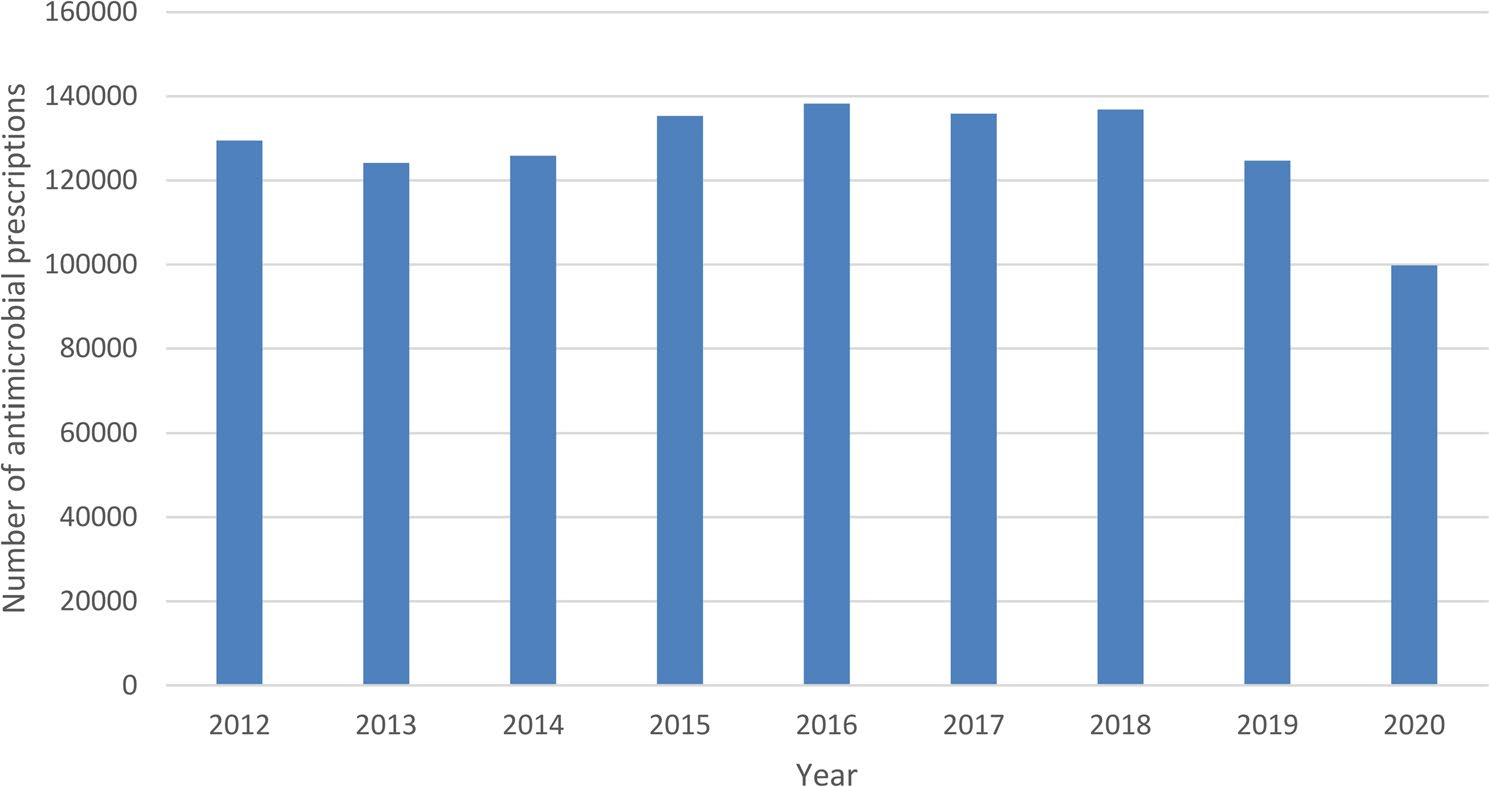
Number of antimicrobial prescriptions per year

In our analyses, we included 1,150,252 antimicrobial prescriptions for 269,574 patients (Table 1). Fourteen PCPs were excluded from the multivariable regression analysis, as data were missing on the total number of registered patients. The average number of antimicrobial prescriptions between the years 2012–2019 was 131,311 per year (range 124,154–138,255). In 2020 there were 99,762 antimicrobial prescriptions, which is a statistically significant decline compared to all previous years (*p* < 0.05) (Fig. 1). A statistically significant difference was found for day of the week, with antimicrobial prescriptions on Monday (242,487) and Friday (240,469) dominating compared to other weekdays, which varied between 194,704 and 211,276 prescriptions.

**Table 1:** Characteristics of study sample

	Antimicrobial prescriptions *n* = 1,150,252	Patients *n* = 269,574
Female gender % (n)	64.6% (743,034)	56.7% (152,714)
Mean age at prescription in years	47.9 years	41.9 years
Age groups in years, % (n)		
0–4	7.0% (80,238)	8.6% (23,268)
5–14	5.7% (65,015)	8.9% (23,904)
15–44	29.9% (344,447)	35.5% (95,827)
45–64	26.6% (306,331)	25.0% (67,481)
65–107	30.8% (354,221)	21.9% (59,094)
With an ICPC code	58.6% (673,909)	NA
Without an ICPC code	41.4% (476,343)	
With an ICPC code related to an infection	50.9% (585,117)	NA
Number of antimicrobial allergies % (n)		
0	98.6% (1,134,169)	99.4% (267,966)
1	1.2% (13,406)	0.5% (1371)
2	0.2% (2247)	0.1% (194)
3 or more	0 (430)	0 (43)
Number of co-morbidities		
0	64.8% (745,910)	76.5% (206,352)
1	26.4% (304,198)	19.6% (52,874)
2	6.9% (79,470)	3.2% (8703)
3 or more	1.8% (20,674)	0.6% (1645)
Migration background % (n)		
Dutch	72.7% (83,5944)	69.3% (186,884)
Morocco	3% (34,846)	3.4% (9098)
Turkey	2.6% (30,084)	2.8% (7503)
Suriname	4.4% (51,037)	4.7% (12,635)
Dutch Caribbean	1.4% (15,805)	1.7% (4466)
Other non-western countries	6.1% (69,687)	7.2% (19,437)
Western countries	9.8% (112,836)	11% (29,541)
Missing	0 (6)	0 (5)
Households with 1 parent	7.8% (89,565)	7.6% (20,589)
Family income		
Low	53.0% (609,228)	49.4% (133,093)
Middle	32.3% (371,795)	39.0% (105,154
High	3.2% (36,755)	2.4% (6536)
Missing	11.5% (132,474)	9.2% (24,791)
Primary care practices size (101 offices)		
Small (*n* = 25)	14.4% (165,921)	13.8% (37,271)
Medium (*n* = 65)	53.3% (612,775)	52.2% (140,730)
Large (*n* = 11)	32.2% (370,254)	33.8% (91,141)
Missing	0.1% (1302)	0.2% (432)
*NA* Not applicable

### Antimicrobial prescriptions according to guideline recommendations

**Fig. 2:**
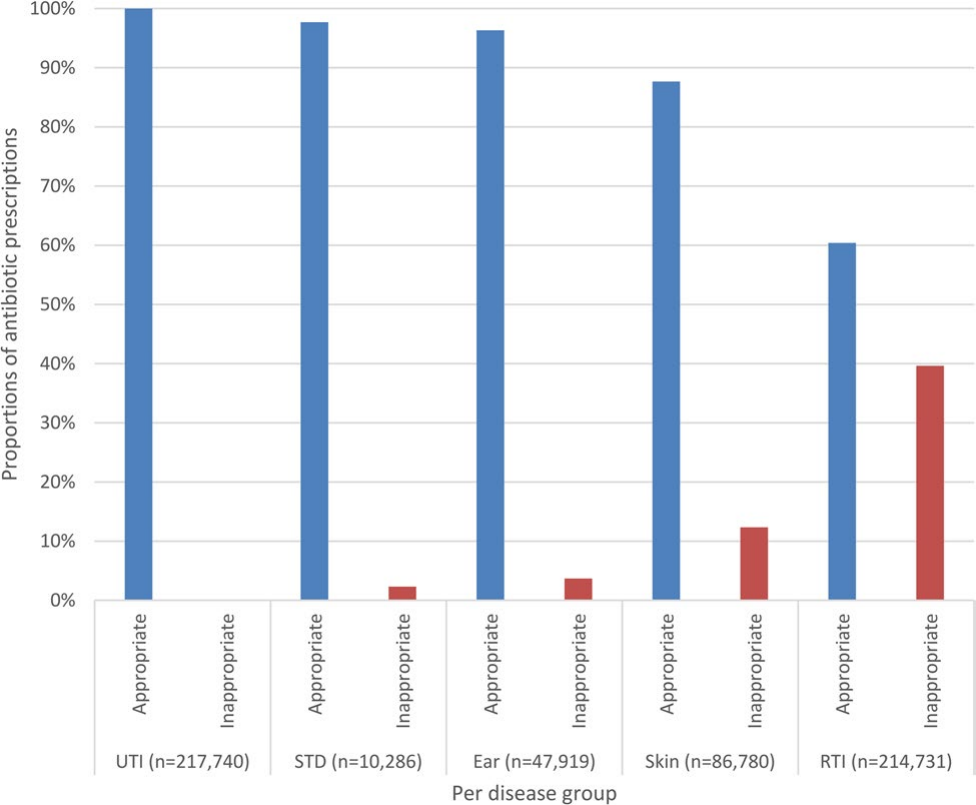
Proportions of appropriate and inappropriate antimicrobial prescriptions per disease group. UTI; Urinary tract infection. STD; Sexual transmitted diseases. Ear; Ear infections. Skin; Skin infections. RTI; Respiratory tract infections

**Fig. 3:**
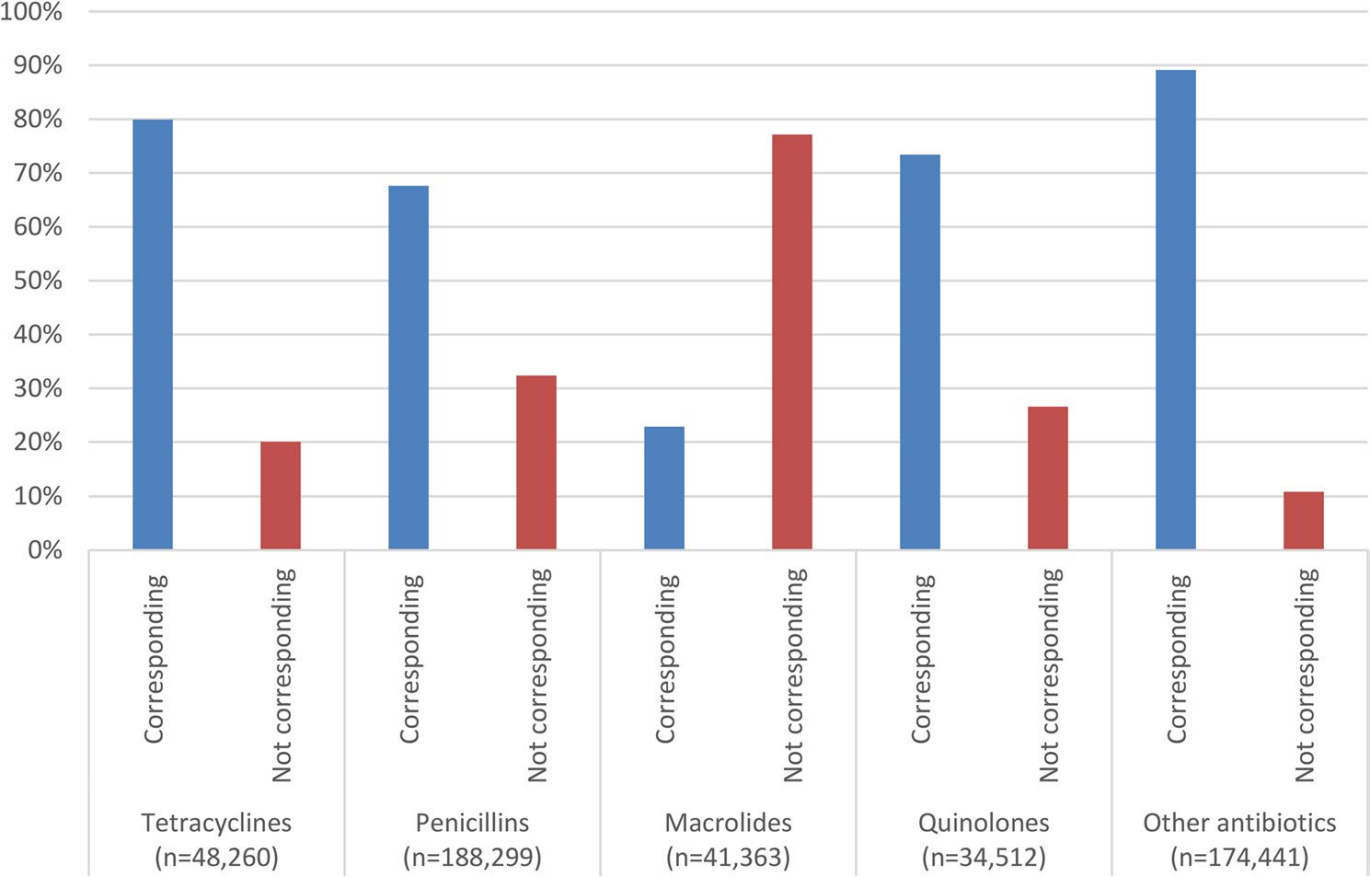
Appropriate antimicrobial prescriptions corresponding with 1st and 2nd choice in guideline

Antimicrobial prescriptions with an ICPC code totalled 673,909, of which 585,117 had an ICPC code corresponding to an infection (Tables 2 and 3). Urinary tract infections (UTI) (37.2%) and RTIs (36.2%) were the most common reason for an antimicrobial prescription. A substantial number of antimicrobial prescriptions were for RTIs and categorized as inappropriate (14.5%). Amongst prescriptions for RTIs alone, 39.6% were classified as inappropriate (Fig. 2). For the 480,792 appropriate antimicrobial prescriptions, 72.3% (347,846) corresponded with guidelines for the 1 st or 2nd choice antimicrobial for the diagnosis. With regard to macrolides, 41,363 appropriate prescriptions were for macrolides, of which over three-quarters (77.1%) were not the 1 st or 2nd choice according to Dutch primary care guidelines (Fig. 3).

**Table 2:** Distribution and characteristics of appropriate and inappropriate antimicrobial prescriptions

	Appropriate antimicrobial prescriptions	Inappropriate antimicrobial prescriptions
Number of antimicrobial prescriptions	480,792	104,325
Female sex % (n)	70.1% (336,910)	61.1% (63,722)
Age groups in years, % (n)		
0–4	8.4% (40,322)	8.6% (9022)
5–14	6.5% (31,279)	6.6% (6895)
15–44	30.0% (144,005)	34.7% (36,208)
45–64	24.7% (118,636)	29.6% (30,904)
65 and older	30.5% (146,550)	20.4% (21,296)
Antimicrobial allergy % (n)		
0	98.6% (474,062)	99.0% (103,240)
1	1.2% (5915)	0.9% (975)
2	0.1% (712)	0.1% (91)
3 or more	0.0% (103)	0.0% (19)
Patients with co-morbidities		
0	66.5% (319,639)	70.3% (73,313)
1	25.4% (122,168)	24.2% (25,213)
2	6.5% (31,268)	4.6%4772
3 or more	1.6% (7717)	1.0%1027
Ethnic background % (n)		
Dutch	75.5% (363,027)	69.4% (72,414)
Moroccan	2.5% (12,087)	3.4% (3538)
Turkish	2.2% (10,458)	3.2% (3336)
Surinamese	3.7% (17,970)	5.2% (5459)
Dutch Caribbean	1.2% (5904)	1.4% (1456)
Global South	5.5% (26,353)	7.5% (7805)
Global North	9.4% (44,988)	9.9% (10,315)
Unknown	0% (1)	0% (2)
Households with 1 parent	12.9% (37,173)	11.9% (8319)
Family income		
Below average income	59.3% (257,008)	57.4% (56,161)
From 1 up to 2 times average income	38.6% (167,506)	40.3% (39,428)
More than 2 times average income	2.1% (9222)	2.3% (2275)
Per disease group % (n)		
UTI	45.3% (217,710)	0.0% (30)
STD	2.1% (10,048)	0.2% (238)
Ear	9.6% (46,154)	1.7% (1765)
GE tract	0.1% (667)	3.1% (3221)
Viral	0.0% (0)	1.6% (1694)
Skin	15.8% (76,069)	10.3% (10,711)
Gyn	0.1% (474)	1.5% (1605)
RTI	27.0% (129,670)	81.5% (85,061)

*UTI* Urinary tract infection, *STD* Sexual transmitted disease, *Ear* Ear infection, *GE* Gastro-intestinal infection, *Gyn* Gynaecological infection, *RTI* Respiratory tract infection

**Table 3:** Number of antimicrobials prescriptions per 1000 patients per size group primary care practice

	Appropriate antimicrobial prescriptions			Inappropriate antimicrobial prescriptions		
Size primary care practice	Mean (95% CI)	SD	Range	Mean (95% CI)	Range	SD
Small	162 (150–173)	27.9	111–205	38 (33–43)	19–64	12.1
Medium	169 (159–180)	41.6	17–270	36 (33–40)	3–87	15.1
Large	154 (128–180)	38.9	86–208	35 (26–44)	15–54	13.2

*CI* Confidence interval, *SD* Standard deviation

.

### Determinants

Female gender, age of five years or older, a Turkish-, Surinamese- or Dutch- Caribbean background, a household with one parent, presence of comorbidities, a medium or large PCP size and Friday as day of prescription, were positively associated with inappropriate antimicrobial prescription (Table 4). A Moroccan migration background was associated with relatively more appropriate antimicrobial prescriptions compared to a Dutch background (Table 4). Determinants associated with inappropriate antimicrobial prescribing for RTIs included male gender, age 5 years or older (except age group 15–44 years), Turkish, Surinamese or Dutch Caribbean background, a low household income, presence of a comorbidity, larger PCP and weekdays other than Friday as day of prescription (Table 5). A check for bias through a multivariable regression analysis that included patients without data in SN data did not show different outcomes (Supplement 5).

**Table 4:** Association of determinants with inappropriate antimicrobial prescribing

	Model 1 OR 95% C.I.	Model 2 OR 95% C.I.		Model 3 OR 95% C.I.	Model 4 OR 95% C.I.
Sex (Female reference)	**0.66 (0.65–0.67)**	**0.67 (0.65–0.68)**		**0.67 (0.65–0.68)**	**0.67 (0.65–0.68)**
Age groups					
0–4 years (reference)	1	1		1	1
5–14 years	**1.40 (1.36–1.43)**	**1.11 (1.06–1.16)**		**1.08 (1.03–1.14)**	**1.08 (1.03–1.14)**
15–44 tears	**1.42 (1.38–1.47)**	**1.12 (1.07–1.17)**		**1.10 (1.05–1.15)**	**1.10 (1.05–1.15)**
45–64 years	**1.76 (1.72–1.79)**	**1.39 (1.33–1.45)**		**1.37 (1.31–1.43)**	**1.37 (1.31–1.43)**
65-and older	**1.81 (1.77–1.84)**	**1.48 (1.41–1.54)**		**1.46 (1.39–1.56)**	**1.46 (1.40–1.52)**
Migration background					
Dutch (reference)		1		1	1
Moroccan		**0.90 (0.87–0.92)**		**0.90 (0.87–0.92)**	**0.89 (0.87–0.92**)
Turkish		**1.15 (1.10–1.21)**		**1.15 (1.10–1.21)**	**1.16 (1.10–1.22)**
Surinamese		**1.25 (1.18–1.31)**		**1.25 (1.19–1.31)**	**1.27 (1.21–1.34)**
Dutch Caribbean		**1.24 (1.19–1.30)**		**1.24 (1.19–1.30)**	**1.24 (1.18–1.29)**
Global South		0.99 (0.93–1.07)		0.99 (0.92–1.07)	0.99 (0.92–1.06)
Global North		**1.16 (1.11–1.21)**		**1.16 (1.11–1.21)**	**1.16 (1.11–1.20)**
Households with 1 parent (2 parents reference)		**1.07 (1.05–1.10)**		**1.07 (1.05–1.10)**	**1.08 (1.05–1.11)**
Household income					
Low (reference)		1		1	1
Middle		0.99 (0.96–1.04)		1.01 (0.96–1.05)	1.00 (0.95–1.04)
High		0.98 (0.94–1.02)		0.98 (0.94–1.02)	0.98 (0.93–1.02)
Number of comorbidities					
0 (reference)				1	1
1				**1.27 (1.11–1.453)**	**1.27 (1.12–1.46)**
2				**1.26 (1.10-1.438)**	**1.26 (1.11–1.45)**
3 or more				1.15 (1.00-1.328)	**1.15 (1.01–1.36)**
Primary Care practice size					
Small (reference)					1
Medium					**1.11 (1.08–1.14)**
Large					**1.03 (1.01–1.05)**
Day of prescription (Friday reference)					**0.96 (0.94–0.98)**

A multivariable logistic regression analysis was conducted in a chronologic order for 4 models to test for an association of determinants with inappropriate antimicrobial prescribing. Model 1 was the first and in an each new model determinants were added.

**Bold** indicates a statistical significant association with inappropriate antimicrobial prescribing (*p* < 0.05)

*OR* Odds ratio, *CI* Confidence Interval

**Table 5:** Association of determinants with inappropriate antimicrobial prescribing for respiratory tract infections

	Model 4 OR (95% C.I.)
Gender (Female as reference)	**1.09 (1.06–1.11)**
Age groups	
0–4 years (reference)	1
5–14 years	**1.27 (1.18–1.33)**
15–44 tears	0.93 (0.88–0.99)
45–64 years	**1.23 (1.16–1.30)**
65-and older	**1.35 (1.80–1.43)**
Migration background	
Dutch (reference)	1
Moroccan	1.00 (0.96–1.04)
Turkish	**1.08 (1.01–1.15)**
Surinamese	**1.25 (1.17–1.33)**
Dutch Caribbean	**1.29 (1.21–1.37)**
Global South	1.07 (0.97–1.18)
Global North	**1.13 (1.07–1.20)**
Households with 1 parent (2 parents reference)	1.01 (0.98–1.05)
Household income	
Low (reference)	1
Middle	**0.87 (0.82–0.93)**
High	**0.92 (0.86–0.97)**
Number of comorbidities	1
0 (reference)	
1	**2.99 (2.56–3.48)**
2	**1.82 (1.56–2.12)**
3 or more	**1.30 (1.10–1.53)**
Primary Care office size	
Small (reference)	1
Medium	**1.17 (1.13–1.21)**
Large	**1.05 (1.02–1.08)**
Day of prescription (Friday reference)	**1.05 (1.02–1.08)**

A multivariable logistic regression analysis was conducted to test the association of determinants with antimicrobial prescribing for respiratory tract infections

**Bold** indicates a statistical significant association with inappropriate antimicrobial prescribing (*p* < 0.05)

*OR* Odds ratio, *CI* Confidence Interval

## Discussion

The primary aim of this study was to identify and to determine the number of antimicrobial prescriptions and determinants of appropriateness in primary care. The principal findings were: (1) the highest rate of antimicrobial inappropriate prescribing, in both number and proportion, was for RTIs, (2) most prescriptions of macrolides did not correspond with the 1 st and 2nd choice in guidelines, and (3) determinants including female gender, age 5 years and older, migration background (Turkish, Surinamese, Dutch-Caribbean), and a large PCP size were all associated with inappropriate antimicrobial prescribing.

### Large registries

A major strength of the study was that we were able to identify novel potential determinants of antimicrobial prescription by combining routine healthcare data with individual socioeconomic - and context data from SN. The use of routine healthcare data for medical research has many advantages, as it provides relatively easy access to rich, ecologically valid, longitudinal data from large populations (67). It potentially more accurately reflects daily practice in accordance with the aim of understanding patterns of daily antimicrobial prescribing in primary care [14].

A potential downside of routinely collected healthcare data is the risk of missing data. The data were not systemically recorded for research but for healthcare purposes. ICPC codes for antimicrobial prescriptions were missing (41,4%) and 13% of the registered ICPC codes were not related to an infection. This registration bias may result in an over- or underestimation of the outcomes. An important limitation is that we were also unable to verify registered diagnoses in this large dataset, which may have led to registration bias, with either under - and over-registration. To better gauge this risk, we compared the study with two prospective Dutch studies on appropriateness of antimicrobial prescribing for RTIs, as prospective data collection is less prone to incorrectly registered or missing data. Both studies had a comparable proportion, at around 40%, of inappropriate antimicrobial prescribing for RTIs [15, 16]. This confirmed the assumption that the large number of antimicrobial prescriptions included in the combined dataset had diluted any potential registration bias and allows us to interpret the findings accordingly. Another limitation is the lack of prescriber characteristics due to privacy concerns. An increase age, a higher years of experience and male gender of the prescribers are associated with more inappropriate antimicrobial prescriptions [10]. This lack of data may have influenced the variable practice size.

These two specific registries (ELAN/SN) have been successfully combined in earlier studies, focussing on cardiovascular risk [7, 17], but this is the first time that the approach has been used for research into AMR. Those earlier studies had methodological issues similar to our study such as missing data and incorrect coding, but nevertheless produced reliable and valid data. Studies of patterns of antimicrobial prescription have been previously conducted using large healthcare registries, but without including socioeconomic data [18, 19].

### Antimicrobial prescribing

The number of antimicrobial prescriptions per year was relatively stable except for the year 2020 (Fig. 1). This significant drop of circa 20% in antimicrobial prescriptions was largely due to the CoViD-19 pandemic, which resulted in relatively fewer bacterial and viral infections and allowed physicians to test their patients before treating them with antimicrobial medication for any presumed bacterial infection [20]. A circa 10% decline in 2020 was noted in the total prescription of antimicrobials in the Netherlands [3], which is broadly similar to the decline in consultations on a national level [21].

RTIs and UTIs were the most common reasons with similar prescription rates for an antimicrobial prescription in our study. Cross-sectional/longitudinal observational studies performed in the United Kingdom also reported RTI and UTI as the most common reason [18, 22], only with relatively fewer prescriptions for UTIs compared to RTIs. Our study showed relatively more antimicrobial prescription for an UTI. The study by Pouwels et al. only included patients with an UTI older than 14 years [18], while UTI’s at a younger age are quite common. The study by Dolk et al. defined ear nose throat infections as a RTI [22].

In both absolute and relative numbers, RTIs in our study accounted for the vast majority of inappropriate antimicrobial prescriptions (81.5%) and within prescriptions for RTIs (39.6%). This number would have been even higher if we had not used a broad definition of appropriate antimicrobial prescribing for an RTI. Prescribing an antimicrobial was considered inappropriate only if the recommendations advised against prescription. It is important to note that Dutch primary care guidelines on RTIs generally advise against prescribing an antimicrobial [23–25]. In two other Dutch studies, one a prospective observational study and the other a pragmatic, cluster-randomized intervention, 46% and 44% of RTI prescriptions, respectively, did not follow guidelines [15, 16].

In our study, only a small proportion of antimicrobial prescriptions for UTIs failed to follow guideline recommendations. This is comparable to a study from the United Kingdom which showed that that 94% of consultations for a UTI led to an antimicrobial prescription within 30 days [18]. Dutch primary care guidelines generally advise treatment of UTI’s with antimicrobials [23].

The prescription of macrolides, that were neither 1st or 2nd guideline choices recommended, was higher than for any other group of antimicrobial compounds. Another Dutch study found similar overprescribing of 2nd choice broad-spectrum antimicrobials [26]. In the Netherlands, macrolides are usually only advised in case of antimicrobial allergy or proven antimicrobial resistance, and they are 1st or 2nd choice antimicrobials for only a few infections. Overprescribing is probably due to the presumed lower burden of use associated with macrolides (fewer daily dosages, shorter courses, less side effects), as most prescriptions in our data were for children below 5 years of age. This is an important area for improvement in primary care antimicrobial prescribing, as macrolides generally have a broader antibacterial spectrum compared to penicillins.

### Determinants

Comorbidity was identified as a determinant of inappropriate antimicrobial prescribing, associations previously reported in several studies [16, 27–30]. Comorbidity is considered a risk factor for severe course of an infection, so a GP may prescribe antimicrobials more readily to prevent more serious complications that might result in hospital admission [25]. The logistic regression did not show a linear dose response relationship. It seems reasonable to posit that patients with multiple comorbidities are at an elevated risk of developing a complicated infection. In such cases, antibiotic treatment is appropriate. To verify this assumption, information on symptoms, outcome of previous infections and clinic judgement of the GP is needed. However, we did not have this information.

Migration background emerged as a determinant with a significant impact on appropriateness of antimicrobial prescribing. While patients with a Moroccan migration background received less inappropriate antimicrobial prescriptions compared to patients with a Dutch background. GPs were found to relatively more often *in*appropriately prescribe antimicrobials for patients with Turkish, Surinamese and Dutch Caribbean backgrounds.

A possible explanation is suggested by a focus group study among patients with a non-Dutch migration background, the expectation of being prescribed an antibiotic by the GP may be higher [31]. GPs presume that patients with an infectious disease want antimicrobial therapy, but fail to actually verify this assumption during shared decision making with the patient [16, 30, 32, 33]. In fact, when asked, patients are usually more worried about the seriousness of their symptoms than eager to be treated [34]. Migrant groups tend to visit their GP more often than people with a non-immigrant Dutch background [35], a higher frequency of GP visits may increase the risk of being prescribed more antimicrobial prescriptions and consequently more inappropriate.

A qualitative study from the Netherlands found no difference in attitudes towards antimicrobials amongst groups with different migration backgrounds compared to the overall Dutch population [36]. However, several different migration backgrounds (Turkish, Moroccan, Surinamese, Syrian and Cape Verdean) were included in this study as one group. Another Dutch study reported that people from a non-Dutch migration background were less knowledgeable about antimicrobials compared to people with a Dutch background [37]. When and how antimicrobials are used in the country of migration background may affect attitudes. For example, in Turkey antimicrobials are used not only for infections but for a broad variety of other diseases and symptoms [38], a pattern that might continue in the Netherlands for patients familiar with both cultures. The higher level of appropriate antimicrobial prescription amongst people with a Moroccan background is likely attributable to lower rates of smoking, which is a known risk factor for complicated RTIs [39]. GPs tend to prescribe antimicrobial medication more easily if there are risk factors for a complicated RTI.

A finding of our study was the association of appropriate antimicrobial prescribing with a PCP size of less than 2,168 patients. Two Canadian studies found a comparable association for practice sizes less than 1,235 or 1,054 patients, respectively [27, 28]. Conversely, a study from the UK reported no association between practice size and appropriate antimicrobial prescribing, although a medium size practice in that study was defined as between 7,928 and 10,941 patients [29]. Differences in practice location and definitions of practice size likely hamper proper comparison between studies. However, the interpretation of the association in our study data is somewhat limited. As there is no dose-relationship in the logistic regression between practice size and the number of inappropriate antimicrobial prescriptions. This may be caused by the lack of prescriber characteristics such as years of working experience and practice characteristics such as number of GPs. A possible explanation for antimicrobial inappropriate prescribing in larger PCPs is that relatively less time per consultation is available, which is independently associated with more antimicrobial inappropriate prescribing [40, 41]. In our study, we interpret practice size as a proxy for continuity of care in daily practice by the same provider. Larger PCPs generally make use of locums, more GPs staffing the practice, and it is known that a higher number of GPs involved with the same population is related to weaker continuity of care in practice. In transition, there is a risk of loss of information essential to adequate follow-up and thus prescribing due to medical uncertainty [42].

Our results also identified the Friday as the weekday prone for (over-)prescribing, in contrast to a UK study that found no differences per weekday [18]. In our case, annex to workload effects, a possible additional explanation might be that GPs use a delayed antimicrobial prescription strategy. In this strategy patients are prescribed antimicrobials before they are actually needed and instructed to collect it, or use it only when specific symptoms worsen. However, this additional supposition would need verification in pharmacy records which we were unable to arrange.

It is important to recognise the distinction between the factors that contribute to inappropriate antimicrobial prescribing for RTIs and those that influence the prescription of antimicrobials for other types of infections. The determinants for RTI’s are male gender, increasing age (except for the 15–44 years age group), a lower household income, and the period from Monday to Thursday. One potential explanation for the observed gender and income disparities is the influence of smoking. Previous research has demonstrated that males and individuals with lower incomes tend to smoke at a higher rate compared to their counterparts, particularly females and those with higher incomes [43]. There is an established association between smoking and an elevated risk of complicated respiratory tract infections (RTIs). It is possible that GPs may be more inclined to prescribe antimicrobials to patients who smoke, given that they may anticipate a more complicated RTI. It should be noted that data on smoking status were unavailable for analysis. The age group between 15 and 44 years old experience fewer RTI episodes than other age groups, but experience more UTIs. Consequently, if they visit a GP less frequently, they have a smaller chance of being prescribed an antimicrobial. The observation that there were fewer inappropriate antimicrobial prescriptions for respiratory tract infections (RTIs) on Fridays was attributed to a lower number of prescriptions for RTIs compared to those for all infections. Conversely, on Mondays, there were significantly more prescriptions for RTIs compared to Fridays. This discrepancy could be attributed to the tendency of patients to seek medical attention after the weekend. They may have waited until the weekend to see if the symptoms of an RTI would resolve spontaneously.

## Conclusion

In our study, we gained new insights and uncovered previously unknown associations with antimicrobial prescription behaviour on patient and practice level. We advise action to improve antimicrobial prescribing especially for RTIs in primary care and explore entries to lower the number of macrolide prescriptions when they are not explicitly needed. As the overarching theme seems to be the availability of time for consultation and shared decision making. We propose that any intervention would benefit from targeted endeavours to reduce practice workload and increase the room for extended consultation time per patient encounter.

## Supplementary Information

Supplementary Material 1. Supplement 1. List of comorbidities.

Supplementary Material 2. Supplement 2. List of inappropriate and appropriate indications for an antimicrobial prescriptions

Supplementary Material 3. Supplement 3. ICPC codes with the recommended antimicrobial according to Dutch primary care guidelines.

Supplementary Material 4. Supplement 4. Flowchart inclusion process antimicrobial prescriptions.

Supplementary Material 5. Supplement 5. Multivariable regression analysis including patients without data in the Statistics Netherlands database.

## Data Availability

A set of coded routine pseudonymized Medical Record data from PCPs that contribute to the ELAN datawarehouse was used for the analysis of antimicrobial prescriptions. This dataset cannot be shared in an open public repository. Medical data in the ELAN datawarehouse are pseudonymized through a not personally traceable patient ID number. However, theoretically patients still can be identified based on gender and date of birth and thus confidentiality is at risk to be violated unintentionally. Patients gave consent to reuse their medical data for the purpose of dedicated and contextually restricted research and quality management, but not in an open and publicly available domain. Data are available upon reasonable request at the ELAN datawarehouse through elanresearch.nl.Data from Statistics Netherlands (SN) is pseudonymized and is only available for researchers through a secure internet connection in a secure environment of SN. All data remains within the secure SN environment. Researchers can apply for access to SN data through www.cbs.nl

## Abbreviations

AMR: Antimicrobial resistance
ATC: Anatomical therapeutic chemical
ELAN: Extramural Leiden Academic Network
EMR: Electronic medical record
ICPC: International classification primary care
LUMC: Leiden university medical centre
PCP: Primary care practice
RTI: Respiratory tract infection
SN: Statistics Netherlands
UTI: Urinary tract infection
