# Supplementary material for: Routine data registries as a basis to analyse and improve the quality of antimicrobial prescription in primary care
Source: BMC Prim Care. 2025 Oct 17;26:318. doi: 10.1186/s12875-025-03008-4 (PMC12532453; doi:10.1186/s12875-025-03008-4)
Supplement: Supplementary file 1 — Supplementary Material 1. Supplement 1. List of comorbidities. [file 12875_2025_3008_MOESM1_ESM.docx]

Supplement 1. List of comorbidities

International Classification of Primary Care (ICPC) codes are used.

- A90 Congenital anomaly nos/multiple (Down syndrome)
- Immunosuppressed, blood forming organs
  - B72 Hodgking diseases
  - B73 Leukaemia
  - B74 Malignant neoplasma blood other
  - B90 HIV-infection/AIDS
- Immunosuppressed, digestive diseases
  - D72 Viral hepatitis
  - D73 Gastroenteritis presumed infection
  - D74 Malignant neoplasm stomach
  - D75 Malignant neoplasm colon/rectum
  - D76 Malignant neoplasm pancreas
  - D77 Malignant digestive neoplasm, other/NOS
  - D94 Chronic enteritis/ulcerative colitis
- Immunosuppressed, cardiovascular diseases
  - K77 Heart failure
- Immunosuppressed, musculoskeletal
  - L71 Malignant neoplasm musculoskeletal
  - L88 Rheumatoid/seropositive arthritis
- Immunosuppressed, neurological
  - N74 Malignant neoplasm nervous system
- Immunosuppressed, lung diseases
  - R83 Other infections airway
  - R89 Congenital anomaly respiratory
  - R91 Bronchiëctasieën
  - R95 COPD
  - R96 Asthma
- Immunosuppressed, urinary tract
  - U75 Malignant neoplasm of kidney
  - U76 Malignant neoplasm of bladder
  - U77 Malignant neoplasm urinary other
  - U85 Congenital anomaly urinary tract
