# Supplementary material for: Routine data registries as a basis to analyse and improve the quality of antimicrobial prescription in primary care
Source: BMC Prim Care. 2025 Oct 17;26:318. doi: 10.1186/s12875-025-03008-4 (PMC12532453; doi:10.1186/s12875-025-03008-4)
Supplement: Supplementary file 2 — Supplementary Material 2. Supplement 2. List of inappropriate and appropriate indications for an antimicrobial prescriptions [file 12875_2025_3008_MOESM2_ESM.docx]

# Supplement 2. List of inappropriate and appropriate indications for an antimicrobial prescriptions

International Classification of Primary Care (ICPC) codes version 1 were used.

## Inappropriate indication for antimicrobial prescription

### Viral disease

- A71 Measles
- A72 Chickenpox
- A74 Rubella
- A76 Other viral disease with exanthema
- A76.01 Exanthema subitum/sixth disease
- A76.02 Erythema infectiosum/fifth disease
- A76.03 Hand-foot-mouth disease
- A77. Other viral disease(s)
- A78. Other infectious disease(s)

### Gastro-intestinal tract infections

- D11 Diarrhoea
- D13 Jaundice
- D22 Worms/other parasites
- D22.01 Enterobiasis
- D22.02 Ascariasis
- D22.03 Taeniasis
- D70 Infectious diarrhoea, dysentery
- D70.01 Salmonella intestinal infection
- D70.02 Shigella/Yersinia/Campylobacter intestinal infection
- D70.03 Giardiasis
- D70.04 Amoebiasis
- D72 Virus hepatitis
- D72.01 Acute hepatitis A
- D73 Suspected gastrointestinal infection
- D83.02 Mouth sores
- D87.01 Gastritis/duodenitis
- D92 Diverticulosis/diverticulitis

### Ear infections

- H72 Otitis media with effusion
- H74 Chronic otitis media/other infection ear
- H74.01 Chronic otitis media

### Respiratory tract infections

- A75 Mononucleosis infectiosa
- D71 Parotitis
- R05 Cough
- R07 Sneezing/nose congestion/runny nose
- R08 Other symptoms/complaints nose
- R09 Symptoms/complaints sinuses (including pain)
- R21 Symptoms/complaints throat
- R21.01 Sore throat
- R22 Symptoms/complaints tonsils
- R71 Whooping cough
- R72 Streptococcal angina/ scarlet fever
- R72.01 Streptococcus angina
- R72.02 Scarlet fever
- R74 Acute upper respiratory tract infection
- R74.01 Common cold
- R74.02 Acute pharyngitis
- R75.01 Acute rhinosinusitis
- R77 Acute laryngitis/tracheitis
- R77.01 Laryngitis subglottica/pseudocroup
- R80 Influenza [ex. R81 Pneumonia ]
- R90 Hypertrophy/chronic infection tonsils/adenoid
- R96.01 Hyperreactivity airways

### Skin

- S10.02 Furunculosis
- S11 Other local infection(s) skin/subcutis
- S11.01 Folliculitis
- S70 Herpes zoster
- S70.01 Herpes zoster
- S71.01 Herpes labialis
- S74 Dermatomycosis(n)
- S74.01 Tinea pedis
- S74.02 Onychomycosis
- S74.03 Pityriasis versicolor
- S75 Moniliasis/candidiasis
- S75.01 Candidiasis mouth/ thrush
- S75.02 Candidiasis nails/ cuticles
- S75.03 Intertrigo/diaper candidiasis
- S76.02 Erythrasma
- S90 Pityriasis rosea

### Urinary Tract Infections

- Y75 Balanitis
- Y75.01 Candida balanitis
- U95 Urolithiasis

### Gynaecology

- X72 Candidiasis urogenital woman
- X84 Vaginitis/vulvitis
- X84.02 Bacterial vaginosis
- W12 Contraception: IUD
- X90 Genital herpes woman

### Sexually Transmitted diseases

- S73 Pediculosis/other skin infection
- S73.02 Pediculosis pubis
- S95 Mollusca contagious
- X91 Condylomata acuminata woman
- Y72 Genital herpes man
- Y76 Condylomata acuminata male

### Skin

- S72 Scabies/other condition caused by mites
- S72.01 Scabies

## Appropriate indication for antimicrobial prescription

### Gastro-intestinal tract infections

- D85 Ulcus duodeni
- D86 Other peptic ulcer
- D86.01 Ulcus ventriculi

### Ear infection

- H04 Secretion from ear
- H05 Blood in/out of ear
- H70 Otitis externa
- H71 Otitis media acuta/myringitis
- H73 Tubular catarrh/tuba stenosis
- H74.02 Mastoiditis

### Respiratory tract infections

- R75 Acute/chronic rhinosinusitis
- R75.02 Chronic rhinosinusitis
- R76 Acute tonsillitis/peritonsillar abscess
- R76.01 Acute tonsillitis
- R76.02 Peritonsillar abscess
- R78 Acute bronchitis/bronchiolitis
- R81 Pneumonia
- R81.01 Legionella pneumonia
- R91 Chronic bronchitis/bronchiectasis
- R91.01 Chronic bronchitis
- R91.02 Bronchiectasis
- R95 Emphysema/COPD
- R96 Asthma
- R96.02 Allergic asthma
- R99.05 Aspiration pneumonia

### Skin

- A78.05 Borreliosis/Lyme
- R73 Furuncle/abscess nose
- S09 Local infection finger/toe/paronychia
- S09.01 Paronychia
- S10 Furuncle/carbuncle/cellulitis locally
- S10.01 Furuncle/carbuncle
- S10.03 Cellulitis [ex. S09]
- S12.01 Tick bite
- S13 Bite human/animal
- S14 Burn/burning skin (any degree)
- S76 Other infection skin/subcutis
- S76.01 Erysipelas
- S84 Impetigo/impetiginisation
- S92.02 Hydradenitis
- S96 Acne
- S96.01 Acne vulgaris
- S96.02 Acne conglobata
- W94 Mastitis puerperalis
- X99.04 Mastitis [ex. W94]

### Urinary Tract infections

- U01 Painful miction
- U02 Frequent micturition/ urge
- U04 Urinary incontinence [ex. P12].
- U04.01 Stress incontinence
- U04.02 Urge incontinence
- U04.03 Mixed incontinence
- U06 Haematuria
- U70 Acute pyelonephritis/pyelitis
- U71 Cystitis/urinary tract infection
- U71.01 Cystitis
- U72 Non-specific urethritis [ex. X99,Y99].
- Y03 Discharge penis/urethra
- Y73 Prostatitis/vesiculitis seminalis
- Y74 Orchitis/epididymitis
- Y74.01 Orchitis
- Y74.02 Epididymitis
- Y75 Balanitis
- W84.01 Urogenital infection in pregnancy

### Gynecology

- W70.01 Endometritis puerperalis
- W94 Mastitis

### Sexually Transmitted diseases

- X13 Blood loss after coitus
- X23 Fear of venereal disease woman
- X70 Lues woman [ex. A90]
- X71 Gonorrhea woman
- X73 Trichomonas urogenital female
- X74 Inflammation of the small pelvis/PID
- X74.01 PID due to Chlamydia
- X84.01 Vaginitis due to Chlamydia
- X85.01 Cervicitis due to Chlamydia
- Y25 Fear of venereal disease man
- Y70 Lues man [ex. A90]
- Y71 Gonorrhea man
- Y99 Other disease(s) genitalia/breasts male
- Y99.03 Chlamydia infection man
