# Supplementary material for: Routine data registries as a basis to analyse and improve the quality of antimicrobial prescription in primary care
Source: BMC Prim Care. 2025 Oct 17;26:318. doi: 10.1186/s12875-025-03008-4 (PMC12532453; doi:10.1186/s12875-025-03008-4)
Supplement: Supplementary file 3 — Supplementary Material 3. Supplement 3. ICPC codes with the recommended antimicrobial according to Dutch primary care guidelines. [file 12875_2025_3008_MOESM3_ESM.docx]

# Supplement 3. ICPC codes with the recommended antimicrobial according to Dutch primary care guidelines

| ICPC code and description | Current 1^e^ and 2nd choice according to guidelines  ATC code Antimicrobial | Previous guidelines  ATC code Antimicrobial | In case of antibiotic allergy ATC code Antimicrobial |
| --- | --- | --- | --- |
| Gastrointestinal tract | | | |
| D85  Ulcus duodeni | J01CA04 Amoxicillin  J01FA09 Clarithromycin |  |  |
| D86  Other peptic ulcer | J01CA04 Amoxicillin  J01FA09 Clarithromycin |  |  |
| D86.01  Ulcus ventriculi | J01CA04 Amoxicillin  J01FA09 Clarithromycin |  |  |
| Ear infections | | | |
| H04  Secretion from ear | J01CF05 Flucloxacillin |  | J01FA10 Azithromycin  J01FA09 Clarithromycin  J01FA01 Erythromycin |
| H05  Blood in/out of ear | J01CF05 Flucloxacillin |  | J01FA10 Azithromycin  J01FA09 Clarithromycin  J01FA01 Erythromycin |
| H70  Otitis externa | J01CF05 Flucloxacillin |  | J01FA10 Azithromycin  J01FA09 Clarithromycin  J01FA01 Erythromycin |
| H71  Otitis media acuta/ myringitis | J01CA04 Amoxicillin |  | J01EE01 Sulfamethoxazole and Trimethoprim  Before 2016  J01EE01 Sulfamethoxazole and Trimethoprim  J01FA10 Azithromycin |
| H73  Tubular catarrh/ tuba stenosis | J01CA04 Amoxicillin |  | J01EE01 Sulfamethoxazole and Trimethoprim  Before 2016  J01EE01 Sulfamethoxazole and Trimethoprim  J01FA10 Azithromycin |
| H74.02  Mastoiditis | J01CA04 Amoxicillin |  | J01EE01 Sulfamethoxazole and Trimethoprim  2016  J01EE01 Sulfamethoxazole and Trimethoprim  J01FA10 Azithromycin |
| R73  Furuncle/ abscess nose | J01CF05 Flucloxacillin |  | J01FA09 Clarithromycin  J01FF01 Clindamycin |
| Respiratory tract infections | | | |
| R75  Acute/chronic rhinosinusitis | J01AA02 Doxycycline  J01CA04 Amoxicillin |  | J01FA01 Erythromycin  J01FA09 Clarithromycin  J01FA10 Azithromycin |
| R75.02  Chronic rhinosinusitis | J01AA02 Doxycycline  J01CA04 Amoxicillin |  | J01FA01 Erythromycin  J01FA09 Clarithromycin  J01FA10 Azithromycin |
| R76  Acute tonsillitis/ peritonsillar abscess | J01CE05 Pheneticillin  J01CE02 Phenoxymethylpenicillin  J01CR02 Amoxicillin-Clavulanicacid |  | J01FA01 Erythromycin  J01FA10 Azithromycin |
| R76.01  Acute tonsillitis | J01CE05 Pheneticillin  J01CE02 Phenoxymethylpenicillin  J01CR02 Amoxicillin-Clavulanicacid |  | J01FA01 Erythromycin  J01FA10 Azithromycin |
| R76.02  Peritonsillar abscess | J01CR02 Amoxicillin-Clavulanicacid |  | J01FA01 Erythromycin  J01FA10 Azithromycin |
| R78  Acute bronchitis/ bronchiolitis | J01AA02 Doxycycline  J01CA04 Amoxicillin |  | J01FA01 Erythromycin  J01FA10 Azithromycin |
| R81  Pneumonia | J01AA02 Doxycycline  J01CA04 Amoxicillin |  | J01FA01 Erythromycin  J01FA10 Azithromycin |
| R81.01  Legionella pneumonia | J01AA02 Doxycycline |  |  |
| R91  Chronic bronchitis/ bronchiectasis | J01AA02 Doxycycline  J01CA04 Amoxicillin |  | J01FA01 Erythromycin  J01FA10 Azithromycin |
| R91.01  Chronic bronchitis | J01AA02 Doxycycline  J01CA04 Amoxicillin |  | J01FA01 Erythromycin  J01FA10 Azithromycin |
| R91.02  Bronchiectasis | J01AA02 Doxycycline  J01CA04 Amoxicillin |  | J01FA01 Erythromycin  J01FA10 Azithromycin |
| R95  Emphysema/ COPD | J01AA02 Doxycycline  J01CA0 |  | J01FA01 Erythromycin  J01FA10 Azithromycin |
| R96  Asthma | J01AA02 Doxycycline  J01CA04 Amoxicillin |  | J01FA01 Erythromycin  J01FA10 Azithromycin |
| R96.02  Allergic asthma | J01AA02 Doxycycline  J01CA04 Amoxicillin |  | J01FA01 Erythromycin  J01FA10 Azithromycin |
| R99.05  Aspiration pneumonia | J01CR02 Amoxicillin-Clavulanicacid |  |  |
| Skin | | | |
| A78.05  Borreliosis/ Lyme | J01AA02 Doxycycline  J01FA10 Azithromycin | Before 2018  J01AA02 Doxycycline  J01CA04 Amoxicillin | Before 2018  J01FA10 Azithromycin |
| S09  Local infection finger/toe/ paronychia | J01CR02 Amoxicillin-Clavulanicacid  J01AA02 Doxycycline |  |  |
| S09.01  Paronychia | J01CR02 Amoxicillin-Clavulanicacid  J01AA02 Doxycycline |  |  |
| S10  Furuncle/ carbuncle/ cellulitis locally | J01CF05 Flucloxacillin  J01FA09 Clarithromycin  J01FF01 Clindamycin  J01FA09 Clarithromycin  J01FA01 Erythromycin  J01FA10 Azithromycin |  |  |
| S10.01  Furuncle/ carbuncle | J01CF05 Flucloxacillin  J01FA09 Clarithromycin  J01FF01 Clindamycin  J01FA09 Clarithromycin  J01FA01 Erythromycin  J01FA10 Azithromycin |  |  |
| S10.03  Cellulitis [ex. S09] | J01CF05 Flucloxacillin  J01FA09 Clarithromycin  J01FF01 Clindamycin  J01FA09 Clarithromycin  J01FA01 Erythromycin  J01FA10 Azithromycin |  |  |
| S12.01  Tick bite | J01AA02 Doxycycline  J01FA10 Azithromycin | Before 2018  J01AA02 Doxycycline  J01CA04 Amoxicillin | Before 2018  J01FA10 Azithromycin |
| S13  Bite human/ animal | J01CR02 Amoxicillin-Clavulanicacid |  | J01AA02 Doxycycline  J01FF01 Clindamycin |
| S14  Burn/burning skin (any degree) | J01CF05 Flucloxacillin |  | J01FA09 Clarithromycin  J01FF01 Clindamycin |
| S76  Other infection skin /subcutis | J01CF05 Flucloxacillin |  | J01FA09 Clarithromycin  J01FF01 Clindamycin |
| S76.01  Erysipelas | J01CF05 Flucloxacillin |  | J01FA09 Clarithromycin  J01FF01 Clindamycin |
| S84  Impetigo/impetiginisation | J01CF05 Flucloxacillin |  | J01FA09 Clarithromycin  J01FF01 Clindamycin  Before 2018  J01FA09 Clarithromycin  J01FF01 Clindamycin  J01FA01 Erythromycin  J01FA10 Azithromycin |
| S92.02 Hydradenitis | J01AA07 Tetracyline |  |  |
| S96  Acne | J01AA02 Doxycycline  J01FA01 Erythromycin  J01AA07 Tetracyline |  |  |
| S96.01  Acne vulgaris | J01AA02 Doxycycline  J01FA01 Erythromycin  J01AA07 Tetracyline |  |  |
| S96.02  Acne conglobata | J01AA02 Doxycycline  J01FA01 Erythromycin  J01AA07 Tetracyline |  |  |
| Urinary tract infections | | | |
| U01  Painful miction | J01XE01 Nitrofurantoin  J01XX01 Fosfomycin  J01EA01 Trimethoprim  J01MA02 Ciprofloxacin  J01CR02 Amoxicillin-Clavulanicacid  J01EE01 Sulfamethoxazole and Trimethoprim | Before 2014  J01MA06 Norfloxacin |  |
| U02  Frequent micturition/ urge | J01XE01 Nitrofurantoin  J01XX01 Fosfomycin  J01EA01 Trimethoprim  J01MA02 Ciprofloxacin  J01CR02 Amoxicillin-Clavulanicacid  J01EE01 Sulfamethoxazole and Trimethoprim | Before 2014  J01MA06 Norfloxacin |  |
| U04  Urinary incontinence [ex. P12]. | J01XE01 Nitrofurantoin  J01XX01 Fosfomycin  J01EA01 Trimethoprim  J01MA02 Ciprofloxacin  J01CR02 Amoxicillin-Clavulanicacid  J01EE01 Sulfamethoxazole and Trimethoprim | Before 2014  J01MA06 Norfloxacin |  |
| U04.01  Stress incontinence | J01XE01 Nitrofurantoin  J01XX01 Fosfomycin  J01EA01 Trimethoprim  J01MA02 Ciprofloxacin  J01CR02 Amoxicillin-Clavulanicacid  J01EE01 Sulfamethoxazole and Trimethoprim | Before 2014  J01MA06 Norfloxacin |  |
| U04.02  Urge incontinence | J01XE01 Nitrofurantoin  J01XX01 Fosfomycin  J01EA01 Trimethoprim  J01MA02 Ciprofloxacin  J01CR02 Amoxicillin-Clavulanicacid  J01EE01 Sulfamethoxazole and Trimethoprim | Before 2014  J01MA06 Norfloxacin |  |
| U04.03  Mixed incontinence | J01XE01 Nitrofurantoin  J01XX01 Fosfomycin  J01EA01 Trimethoprim  J01MA02 Ciprofloxacin  J01CR02 Amoxicillin-Clavulanicacid  J01EE01 Sulfamethoxazole and Trimethoprim | Before 2014  J01MA06 Norfloxacin |  |
| U06  Haematuria | J01XE01 NITROFURANTOIN J01XX01 FOSFOMYCIN  J01EA01 TRIMETHOPRIM  J01MA02 CIPROFLOXACIN  J01CR02 Amoxicillin-Clavulanicacid  J01EE01 Sulfamethoxazole and Trimethoprim | Before 2014  J01MA06 Norfloxacin |  |
| U70  Acute pyelonephritis/pyelitis | J01MA02 CIPROFLOXACIN  J01CR02 Amoxicillin-Clavulanicacid  J01EE01 Sulfamethoxazole and Trimethoprim | Before 2014  J01MA06 Norfloxacin |  |
| U71  Cystitis/ urinary tract infection | J01XE01 Nitrofurantoin  J01XX01 Fosfomycin  J01EA01 Trimethoprim  J01MA02 Ciprofloxacin  J01CR02 Amoxicillin-Clavulanicacid  J01EE01 Sulfamethoxazole and Trimethoprim | Before 2014  J01MA06 Norfloxacin |  |
| U71.01  Cystitis | J01XE01 Nitrofurantoin  J01XX01 Fosfomycin  J01EA01 Trimethoprim  J01MA02 Ciprofloxacin  J01CR02 Amoxicillin-Clavulanicacid  J01EE01 Sulfamethoxazole and Trimethoprim | Before 2014  J01MA06 Norfloxacin |  |
| U72  Non-specific urethritis [ex. X99,Y99]. | J01XE01 Nitrofurantoin  J01XX01 Fosfomycin  J01EA01 Trimethoprim  J01MA02 Ciprofloxacin  J01CR02 Amoxicillin-Clavulanicacid  J01EE01 Sulfamethoxazole and Trimethoprim | Before 2014  J01MA06 Norfloxacin |  |
| W84.01 | J01XE01 Nitrofurantoin  J01XX01 Fosfomycin  J01EA01 Trimethoprim  J01MA02 Ciprofloxacin  J01CR02 Amoxicillin-Clavulanicacid  J01EE01 Sulfamethoxazole and Trimethoprim |  |  |
| Y03  Discharge penis/urethra | J01DD04 Cefrtriaxone  J01MA02 Ciprofloxacin  J01CA04 Amoxicillin  J01FA10 Azithromycin |  |  |
| Y73  Prostatitis/vesiculitis seminalis | J01MA02 Ciprofloxacin  J01CR02 Amoxicillin-Clavulanicacid  J01EE01 Sulfamethoxazole and Trimethoprim |  |  |
| Y74  Orchitis/epididymitis | J01XMA12 Levofloxacin  J01XMA01 Ofloxacin  J01EE01 Sulfamethoxazole and Trimethoprim  J01AA02 Doxycycline |  |  |
| Y74.01  Orchitis | J01XMA12 Levofloxacin  J01XMA01 Ofloxacin  J01EE01 Sulfamethoxazole and Trimethoprim  J01AA02 Doxycycline |  |  |
| Y74.02  Epididymitis | J01XMA12 Levofloxacin  J01XMA01 Ofloxacin  J01EE01 Sulfamethoxazole and Trimethoprim  J01AA02 Doxycycline |  |  |
| Y75  Balanitis | J01FA09 Clarithromycin  J01CR02 Amoxicillin-Clavulanicacid |  |  |
| Gynecology | | | |
| W70.01  Endometritis puerperalis | J01CA04 Amoxicillin |  |  |
| W94  Mastitis puerperalis | J01CF05 Flucloxacillin |  | J01FA01 Erythromycin |
| X99.04  Mastitis [ex. W94] | J01CF05 Flucloxacillin |  | J01FA01 Erythromycin |
| Sexual transmitted diseases | | | |
| X13  Blood loss after coitus | J01AA02 Doxycycline  J01FA10 Azithromycin  J01CA04 Amoxicillin |  |  |
| X23  Fear of venereal disease woman | J01AA02 Doxycycline  J01FA10 Azithromycin  J01CA04 Amoxicillin |  |  |
| X70  Lues woman [ex. A90] | J01CE08 Benzylpenicillin |  | J01AA02 Doxycycline |
| X71  Gonorrhea woman | J01DD04 Ceftriaxone  J01MA02 Ciprofloxacin  J01CA04 Amoxicillin  J01FA10 Azithromycin |  |  |
| X73  Trichomonas urogenital female | J01XD01 Metronidazole  J01FF01 Clindamycin |  |  |
| X74  Inflammation of the small pelvis/PID | J01XMA01 Ofloxacin  J01XMA12 Levofloxacin  J01AA02 Doxycycline  J01XD01 Metronidazole  J01DD04 Ceftriaxone |  |  |
| X74.01  PID due to Chlamydia | J01XMA01 Ofloxacin  J01XMA12 Levofloxacin  J01AA02 Doxycycline  J01XD01 Metronidazole  J01DD04 Cefrtriaxone |  |  |
| X84.01  Vaginitis due to Chlamydia | J01AA02 Doxycycline  J01FA10 Azithromycin  J01CA04 Amoxicillin |  |  |
| X85.01  Cervicitis due to Chlamydia | J01AA02 Doxycycline  J01FA10 Azithromycin  J01CA04 Amoxicillin |  |  |
| Y25  Fear of venereal disease man | J01FA10 Azithromycin  J01AA02 Doxycycline |  |  |
| Y70  Lues man [ex. A90] | J01CE08 Benzylpenicillin  J01AA02 Doxycycline |  |  |
| Y71  Gonorrhea man | J01DD04 Cefrtriaxone  J01MA02 Ciprofloxacin  J01CA04 Amoxicillin  J01FA10 Azithromycin |  |  |
| Y99  Other disease(s) genitalia/breasts male | J01FA10 Azithromycin  J01AA02 Doxycycline |  |  |
| Y99.03  Chlamydia infection man | J01FA10 Azithromycin  J01AA02 Doxycycline |  |  |

NHG: Dutch General Practitioner society
