# Supplementary material for: Routine data registries as a basis to analyse and improve the quality of antimicrobial prescription in primary care
Source: BMC Prim Care. 2025 Oct 17;26:318. doi: 10.1186/s12875-025-03008-4 (PMC12532453; doi:10.1186/s12875-025-03008-4)
Supplement: Supplementary file 4 — Supplementary Material 4. Supplement 4. Flowchart inclusion process antimicrobial prescriptions. [file 12875_2025_3008_MOESM4_ESM.docx]

# Supplement 4. Flowchart inclusion process antimicrobial prescriptions

Antimicrobial prescriptions removed

Duplicate antimicrobial prescriptions (n =2,460,196)

Antimicrobial prescription not by GP (n =184,058)

Not oral antimicrobial prescriptions (n =4484)

All identified antimicrobial prescriptions between 2012 and 2021 in ELAN datawarehouse

N = 4,145,199

**Identification**

Antimicrobial prescriptions linked with CBS microdata

N = 1,496,461

Reasons for exclusions:

Prophylactic (n = 122,659)

Prescribed in the year 2021 (n =79,418 )

No linkage with CBS micro data possible (n = 144,132)*

**Linked with CBS microdata**

Analysed antimicrobial prescriptions

N =1,150,252

**Included**

*Linkage was not possible for 35,321 patients. Statistics Netherlands does not collect data for people who stay for a short period in the Netherlands or do not have a social security number.
