# Supplementary material for: Routine data registries as a basis to analyse and improve the quality of antimicrobial prescription in primary care
Source: BMC Prim Care. 2025 Oct 17;26:318. doi: 10.1186/s12875-025-03008-4 (PMC12532453; doi:10.1186/s12875-025-03008-4)
Supplement: Supplementary file 5 — Supplementary Material 5. Supplement 5. Multivariable regression analysis including patients without data in the Statistics Netherlands database. [file 12875_2025_3008_MOESM5_ESM.docx]

# Supplement 5. Multivariable regression analysis including patients without data in Statistics Netherlands database

|  | Model 4 OR (95% CI) |
| --- | --- |
| Sex (Female as reference) | **0.67 (0.65-0.68)** |
| Age groups  0 – 4 years (reference)  5-14 years  15-44 tears  45-64 years  65 years and older | 1  **1.08 (1.03-1.14)**  **1.10 (1.05 -1.15)**  **1.37 (1.31-1.43)**  **1.46 (1.40-1.52)** |
| Migration background  Dutch (reference)  Moroccan  Turkish  Surinamese  Dutch Caribbean  Global South  Global North | 1  **0.89 (0.87-0.92)**  **1.16 (1.10-1.22)**  **1.27 (1.21-1.34)**  **1.24 (1.18-1.2**9)  0.99 (0.92-1.06)  **1.16 (1.11-1.20)** |
| Households with 1 parents (2 parents reference) | **1.08 (1.05-1.11)** |
| Household income  Low (reference)  Middle  High | 1  1.00 (0.951-1.06)  0.99 (0.93 -1.04) |
| Number of comorbidities  0 (reference)  1  2  3 or more | 1  **1.28 (1.12-1.46)**  **1.27 (1.11-1.45)**  **1.16 (1.01-1.36)** |
| Primary Care practice size  Small (reference)  Medium  Large | 1  **1.11 (1.08-1.14)**  **1.03 (1.01-1.05)** |
| Weekday of prescription (Friday reference) | **0.96 (0.94-0.98)** |

OR; Odds Ratio. C.I.; Confidence Interval. GP; General practitioner.
